# Is Red Cell Distribution Width a Novel Biomarker of Breast Cancer Activity? Data From a Pilot Study

**DOI:** 10.4021/jocmr1214w

**Published:** 2013-02-25

**Authors:** Charalampos Seretis, Fotios Seretis, Emmanouil Lagoudianakis, George Gemenetzis, Nikolaos S. Salemis

**Affiliations:** a Breast Surgery Unit, 401 General Army Hospital of Athens, Greece; b Postgraduate Programme of Haemostasis - Transfusion Therapy, Attikon University Hospital, Medical School of Athens, Greece

**Keywords:** Breast, Cancer, Biomarker, Red cell distribution width

## Abstract

**Background:**

Red cell distribution width (RDW) is a parameter of the standard full blood count tests, measuring the size variability of erythrocytes. Recently, its elevation has been proven to reliably reflect the extent systematic inflammation, mainly in cardiometabolic diseases. Up to date, its association with solid malignancies has been scarcely investigated.

**Methods:**

We performed a retrospective study, in order to examine if RDW values comparing elevation is correlated with the histopathological parameters of breast cancer (tumor size, grade, lymphatic spread, overexpression of hormonal receptors and HER2 protein), as well as to assess the existence of any differences in RDW comparing two age-matched groups of patients with benign and malignant breast lesions respectively.

**Results:**

RDW was significantly higher in patients with breast cancer, when compared to the enrolled patients with fibroadenomas. Moreover, in the breast cancer group, RDW elevation was significantly correlated with larger primary tumors, higher number of infiltrated axillary lymph nodes and HER2 overexpression, while it was inversely associated with the tumor grade.

**Conclusions:**

Our pilot study demonstrated tha Red cell distribution width may be a novel biomarker of the activity of breast cancer. Although our preliminary findings need to be evaluated by studies with larger samples of patients, based on commonly accepted pathophysiological principles, we presume that they will be applicable not only in breast cancer, but also in other types of solid cancers, providing a simple and cost-effective biomarker of cancer surveillance.

## Introduction

Red cell distribution with (RDW) is a widely used laboratory parameter for the quantification of the extent of erythrocyte anisocytosis, which reflects the variability of the size of the circulating erythrocytes [1]. Its main clinical application has until recently been limited to the discrimination of iron deficiency anemia from thalassemia trait, as well as a marker of excluding iron deficiency anemia in cases where serum ferritin does not accurately indicate the total iron store [2, 3]. However, fluctuation of RDW has been reported in many pathophysiological conditions, which spread across a far wider spectrum, with RDW elevation being firmly associated with ischemic heart disease, acute and chronic heart failure, atherosclerosis, vascular occlusive disease, hypertension, active status of inflammatory bowel disease, rheumatoid arthritis and, in general, with conditions leading to a progressive inflammatory status [4-8]. The molecular basis of the above mentioned associations has been mainly attributed to the ability of RDW to reliably reflect an increase of the levels of circulating cytokines, such as IL-6, TNF-a and hepcidin [9, 10].

Despite the fact that cancer is widely accepted to stand as both a cause and a result of chronic inflammation [11, 12], RDW elevation has scarcely been investigated as a potential biomarker of solid cancer activity [13-17], with no studies assessing RDW in breast cancer, with the exception of a single study which demonstrated that RDW was significantly correlated with bone marrow metastatic spread in a sample of breast cancer patients [18]. Our study is the first systematic one to evaluate whether RDW elevation may have a potential role as a biomarker of breast cancer activity, examining the presence of any associations between RDW and histopathological parameters in breast cancer patients, as well as to assess the existence of any differences in RDW comparing patients with benign and malignant breast lesions.

## Patients and Methods

To test our hypothesis, we performed a retrospective study, in which were enrolled 49 women with breast neoplasias, who were surgically treated in our Department, consisting of 14 patients with fibroadenomas (Group A) and 35 with breast cancer (Group B). Based upon the preoperative diagnosis, the patients were submitted either to local excision in cases of benign disease and modified radical mastectomy in cases of breast cancer. Concerning the breast cancer group, in order to standardize the oncological profile of the tumors, only patients with unifocal grade II (n = 18) and grade III (n = 17) invasive ductal carcinoma were included. The rest of the other exclusion criteria of the study were the presence of hematological disorders, active inflammation, anemia (assessed through preoperative measurement of hemoglobin), iron supplementation therapy, recent venous thrombosis (past 6 months) and recent blood transfusion (past 3 months). Each patient’s medical record was reviewed independently by two physicians of our Department, in order to ensure that exclusion criteria were accurately met in every patient candidate of being enrolled in our study. Red cell distribution width (RDW) values were calculated using automatic analysis in the framework of the routine pre-operative full blood count test (normal range: 11-14.6%). The blood samples were obtained in the morning before the scheduled operation, between 07.30 - 09.00, in order to standardize the impact of circulating hormones (circadian rhythm) on the number and subtype distribution of the various blood cell indices. Moreover, the blood samples obtained were fasting, in accordance to the Department’s protocol for routine pre-operative evaluation of the patients scheduled for elective surgery, which is applied for the standardization of the preoperative values of biochemical tests.

We assessed the existence of differences in RDW values regarding the underlying breast pathology (benign or malignant) and, concerning the breast cancer group, we examined the presence of any correlations between RDW and the number of infiltrated axillary lymph nodes and the size of the primary tumor. Moreover, we examined the presence of statistically significant differences of RDW values with respect to the over-expression of estrogen and progesterone receptors, as well as the over-expression of HER2 protein.

Our statistical analyses were performed using SPSS 16.0 software package. All continuous and categorical variables were assessed for the values’ normal distribution. Continuous variables were correlated using independent samples t-test, while chi-square was used when comparing categorical variables. A P < 0.05 level was considered statistically significant.

## Results

**Figure 1:**
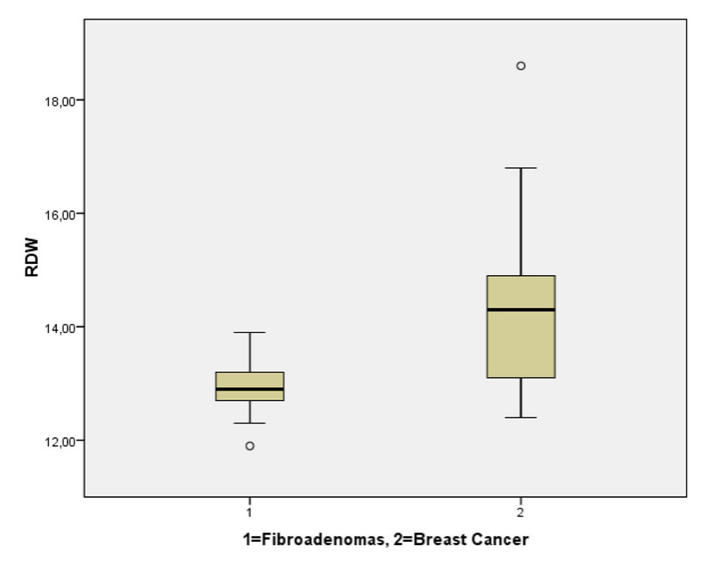
Comparative distribution of RDW in the patients with fibroadenomas and breast cancer. RDW was significantly higher in cases of underlying malignancy (P < 0.001).

There were no statistically significant differences of the mean age and age span between the group of patients with fibroadenomas and the patients with breast cancer (Group A versus Group B). However, during this initial categorization (benign or malignant lesion), RDW was significantly higher in the breast cancer cohort (P < 0.001, Group A versus Group B). More specifically, mean age was 60.1 ± 6.3 years in Group A and 60.5 years ± 13.75 for Group B, while mean RDW was 12.9 ± 0.5 for the patients with benign breast lesions (Group A) and 14.3 ± 1.46 for the breast cancer patients (Group B). The distribution of RDW values after this initial classification of our patients (benign versus malignant) are displayed in Figure 1.

**Figure 2:**
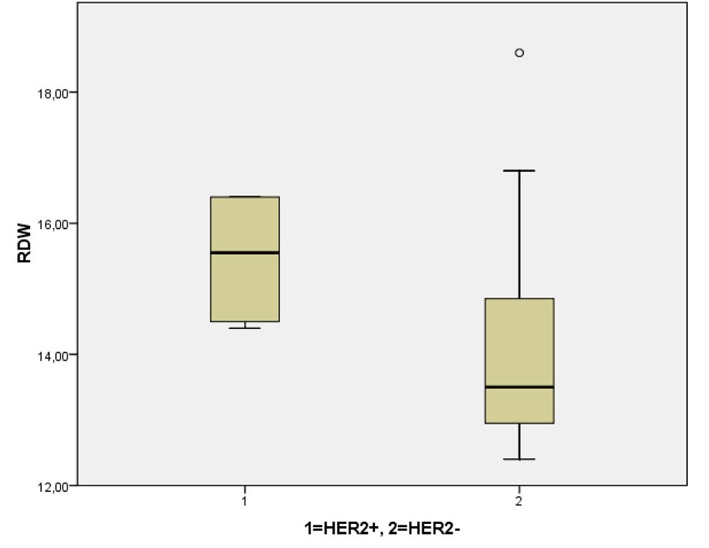
Red cell distribution width with respect to the over-expression of HER2 protein: RDW were significantly higher in cases of HER2 over-expression.

**Figure 3:**
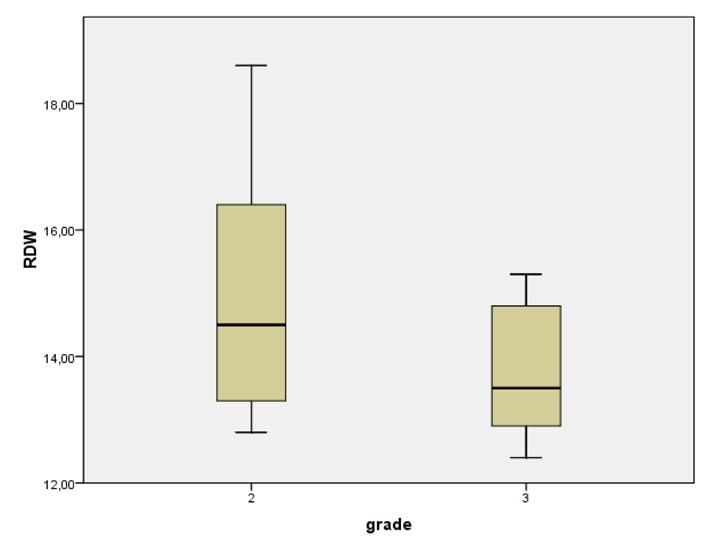
Red cell distribution width with respect to the grade of the cancerous lesions: RDW was significantly higher in patients with grade II cancer.

**Figure 4:**
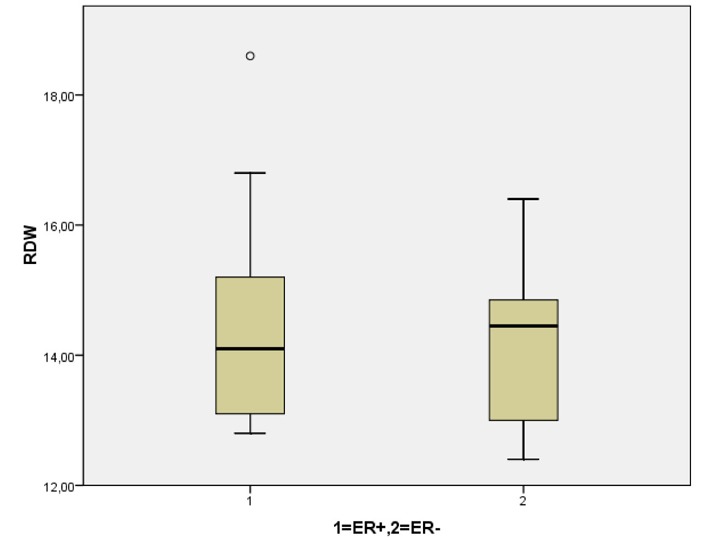
Red cell distribution width with respect to the over-expression estrogen receptors (P = ns).

**Figure 5:**
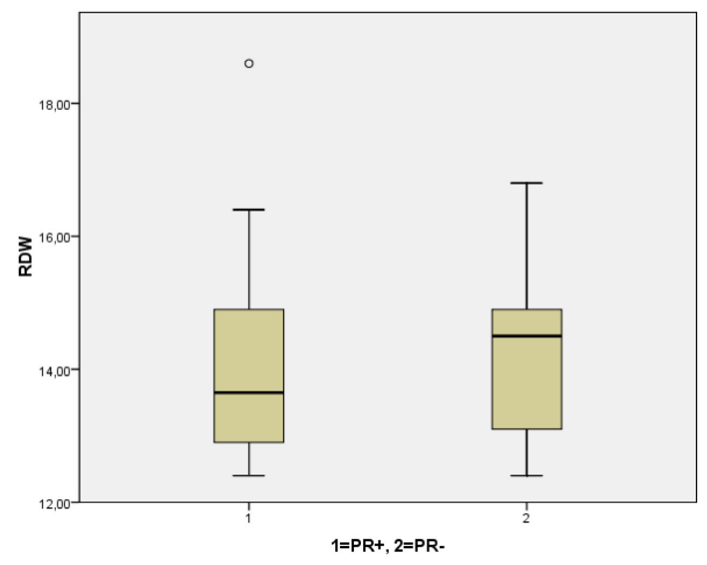
Red cell distribution width with respect to the over-expression progesterone receptors (P = ns).

With respect of the breast cancer patients, mean tumor diameter was 22.89 mm (SD: 13), as well as the mean number of infiltrated lymph nodes was 2.3 (SD: 4.86). Patients with grade III tumors had more extended lymphatic spread (4.1 ± 6.5 infiltrated nodes in grade III cancer versus 0.6 ± 0.78 in grade II, P = 0.01) and larger tumors (28.3 ± 13.8 mm in grade III cancer versus 18 ± 10.35 in grade II, P < 0.05). Concerning the immunohistochemical profile of the cancerous lesions, over-expression of estrogen receptors (ER+) and progesterone receptors (PR+) occurred in 19/35 (54.2%) and 16/35 (45.7%) patients respectively, while 8/35 tumors were stained positive for HER2 protein over-expression (22.9%). Statistical analysis revealed that elevated RDW was significantly correlated with older age (P < 0.001), total number of infiltrated lymph nodes (P < 0.001), tumor diameter (P < 0.01) and HER2 over-expression (P < 0.01) (Fig. 2). Moreover, our results demonstrated that patients with grade II carcinoma had a significantly higher RDW mean value compared to the patients with grade III carcinoma (P = 0.015) (Fig. 3). No statistically significant differences for RDW values were revealed when the cancer patients were categorized according to the positivity for hormonal (both estrogen and progesterone) receptors expression (Fig. 4, 5) respectively.

## Discussion

Red cell distribution width (RDW) is a widely available by the vast majority of automated analysts. Reflecting the size heterogeneity of the circuiting erythrocytes, higher RDW values are suggestive of increased variation of red cell volumes (anisocytosis). Until recently, RDW had been a usually overlooked erythrocyte parameter, however, recent studies have clearly demonstrated that RDW is a reliable biomarker of cardiovascular morbidity and mortality, as well as an indicator of active, acute or chronic inflammatory conditions, such as infections and auto-immune diseases, since RDW is an early detector of iron deficiency anemia, impaired iron mobilization, increased oxidative stress status and its elevation has been firmly associated with elevation of other inflammation markers, such as C-reactive protein, Interleukin-6 and Tumor Necrosis Factor-a [6, 19].

Despite the great number of published studies evaluated the clinical significance of RDW as a prognostic factor in patients with impaired cardiometabolic function and active inflammation, extremely limited data exist concerning the potential consideration of RDW as a biomarker of cancer growth and metastatic activity in solid malignancies. To the best of our knowledge, there are only two studies assessing the utility of RDW as an additional factor for increasing the diagnostic accuracy of anemia as a screening method in colorectal cancer [16, 17], demonstrating conflicting results. Moreover, a recent study by Beyzit et al [14] indicated that elevated RDW could be a useful biomarker in order to discriminate benign from malignant causes of biliary obstruction, with a sensitivity of 72% and specificity of 69%, using 14.8% as a cut-off value for RDW. Apart from the above mentioned, there are two other published studies, by Ozkalemkas et al and Seitanides et al, suggesting that elevated RDW was significantly correlated with disseminated solid malignancies to the bone marrow, with the study by Seitanides et al being the one including patients with breast cancer as the primary tumor, without providing, though, any other data concerning a possible correlation of RDW with the histopathological parameters of the breast lesions [15, 18]. Finally, in a study by Baicus et al, RDW was significantly elevated in a cohort of patients with various types of malignancies, when compared to non-cancer patients [13]. Thus, it is evident that our study is one of the very few to investigate whether RDW elevation may be of any significance with respect to the biological behavior of solid malignancies and is the first one to document a statistically significant correlation between RDW and principle histopathological parameters in breast cancer, even in the framework of a pilot study.

Our preliminary results demonstrated that elevated RDW could be helpful in the differential diagnosis of the nature of a breast tumor -benign or malignant-, being significantly higher in the group of patients with breast cancer. However, it is obvious that RDW cannot be regarded as a specific detector of malignancy. As the exclusion criteria of our study highlight, RDW value can be influenced in many common pathological conditions. Also, taking into account the relatively small sample of the patients enrolled in our study, it would be rather endangered to propose a cut-off value of RDW that would be more safely associated with underlying malignancy. Nevertheless, we should take into account that RDW is always available in routine blood tests and does not increase the cost of diagnostic strategy; this particular point stands for a major advantage regarding its easy and cost-effective application.

Without a doubt, one of the most interesting findings of our study was the fact that although higher values of RDW were significantly correlated with the primary tumor diameter and the absolute number of the infiltrated axillary lymph nodes, RDW was inversely associated with the tumor grade (significantly higher in patients with grade II invasive ductal carcinomas than in patients with grade III cancer). This particular finding appears to be a controversy to the rationale of elevation of RDW according to the presence of a more active inflammatory process, as a higher tumor grade generally enhances the local and systematic inflammatory reaction [20]. However, at least theoretically, a possible explanation could be that more aggressive tumors demonstrate features that help them “escape” from immuno-surveillance and consequently may not trigger an extended inflammatory reaction during their progression.

Another intriguing finding of our pilot study was the firm association of RDW elevation and HER overexpression. Indeed, HER2 overexpression is regarded to lead to a significant upregulation of IL-6, which plays a crucial role in the promotion of the tumor-immune system interactions, the establishment of a generalized inflammatory status along with the progression of cancer and the enhancement of local growth and metastatic spread [21]. From this point of view, RDW could also be a biomarker for monitoring the response in cases of implementation of anti-HER2 agents, comparing baseline measurements before the initiation of the treatment with sequential measurements according to the therapeutic protocols.

Summarizing, our study demonstrated the Red cell distribution width may be a novel biomarker of the activity of breast cancer. Although our preliminary findings need to be evaluated by larger studies, the close interplay between inflammation and cancer provides a solid theoretical base to support the evidence provided by this pilot study.

## References

[1] Forhecz Z, Gombos T, Borgulya G, Pozsonyi Z, Prohaszka Z, Janoskuti L (2009). Red cell distribution width in heart failure: prediction of clinical events and relationship with markers of ineffective erythropoiesis, inflammation, renal function, and nutritional state. Am Heart J.

[2] Demir A, Yarali N, Fisgin T, Duru F, Kara A (2002). Most reliable indices in differentiation between thalassemia trait and iron deficiency anemia. Pediatr Int.

[3] van Zeben D, Bieger R, van Wermeskerken RK, Castel A, Hermans J (1990). Evaluation of microcytosis using serum ferritin and red blood cell distribution width. Eur J Haematol.

[4] Gunebakmaz O, Kaya MG, Duran M, Akpek M, Elcik D, Eryol NK (2012). Red Blood Cell Distribution Width in 'Non-Dippers' versus 'Dippers'. Cardiology.

[5] Nishizaki Y, Yamagami S, Suzuki H, Joki Y, Takahashi S, Sesoko M, Yamashita H (2012). Red blood cell distribution width as an effective tool for detecting fatal heart failure in super-elderly patients. Intern Med.

[6] Karabulut A, Uzunlar B (2012). Correlation between red cell distribution width and coronary ectasia in the acute myocardial infarction. Clin Appl Thromb Hemost.

[7] Yesil A, Senates E, Bayoglu IV, Erdem ED, Demirtunc R, Kurdas Ovunc AO (2011). Red cell distribution width: a novel marker of activity in inflammatory bowel disease. Gut Liver.

[8] Lee WS, Kim TY (2010). Relation between red blood cell distribution width and inflammatory biomarkers in rheumatoid arthritis. Arch Pathol Lab Med.

[9] de Gonzalo-Calvo D, de Luxan-Delgado B, Rodriguez-Gonzalez S, Garcia-Macia M, Suarez FM, Solano JJ, Rodriguez-Colunga MJ (2012). Interleukin 6, soluble tumor necrosis factor receptor I and red blood cell distribution width as biological markers of functional dependence in an elderly population: a translational approach. Cytokine.

[10] Rhodes CJ, Howard LS, Busbridge M, Ashby D, Kondili E, Gibbs JS, Wharton J (2011). Iron deficiency and raised hepcidin in idiopathic pulmonary arterial hypertension: clinical prevalence, outcomes, and mechanistic insights. J Am Coll Cardiol.

[11] Chiba T, Marusawa H, Ushijima T (2012). Inflammation-associated cancer development in digestive organs: mechanisms and roles for genetic and epigenetic modulation. Gastroenterology.

[12] Mladenova D, Kohonen-Corish MR (2012). Review: Mouse models of inflammatory bowel disease--insights into the mechanisms of inflammation-associated colorectal cancer. In Vivo.

[13] Baicus C, Caraiola S, Rimbas M, Patrascu R, Baicus A (2011). Utility of routine hematological and inflammation parameters for the diagnosis of cancer in involuntary weight loss. J Investig Med.

[14] Beyazit Y, Kekilli M, Ibis M, Kurt M, Sayilir A, Onal IK, Purnak T (2012). Can red cell distribution width help to discriminate benign from malignant biliary obstruction? A retrospective single center analysis. Hepatogastroenterology.

[15] Ozkalemkas F, Ali R, Ozkocaman V, Ozcelik T, Ozan U, Ozturk H, Kurt E (2005). The bone marrow aspirate and biopsy in the diagnosis of unsuspected nonhematologic malignancy: a clinical study of 19 cases. BMC Cancer.

[16] Spell DW, Jones DV Jr., Harper WF, David Bessman J (2004). The value of a complete blood count in predicting cancer of the colon. Cancer Detect Prev.

[17] Speights VO, Johnson MW, Stoltenberg PH, Rappaport ES, Helbert B, Riggs MW (1992). Complete blood count indices in colorectal carcinoma. Arch Pathol Lab Med.

[18] Seitanides B, Giakoumakis G, Tsakona C (1988). Increased red cell volume distribution width in patients with bone marrow metastases. J Clin Pathol.

[19] Agarwal S (2012). Red cell distribution width, inflammatory markers and cardiorespiratory fitness: Results from the National Health and Nutrition Examination Survey. Indian Heart J.

[20] Lee AH, Happerfield LC, Bobrow LG, Millis RR (1997). Angiogenesis and inflammation in invasive carcinoma of the breast. J Clin Pathol.

[21] Hartman ZC, Yang XY, Glass O, Lei G, Osada T, Dave SS, Morse MA (2011). HER2 overexpression elicits a proinflammatory IL-6 autocrine signaling loop that is critical for tumorigenesis. Cancer Res.

